# Dynamic confinement controls the porous-to-free convection transition

**DOI:** 10.1073/pnas.2533675123

**Published:** 2026-05-28

**Authors:** Dario M. Schwendener, Jerome Noir, Jonas Latt, Christophe Coreixas, Xiang-Zhao Kong

**Affiliations:** ^a^ Department of Earth and Planetary Sciences, Institute of Geophysics, Geothermal Energy and Geofluids Group, ETH Zürich, Zurich 8092, Switzerland; ^b^ Department of Earth and Planetary Sciences, Institute of Geophysics, Earth and Planetary Magnetism, Institute of Geophysics, ETH Zürich, Zurich 8092, Switzerland; ^c^ Department of Computer Science, University of Geneva, Carouge 1227, Switzerland; ^d^ Institute for Advanced Study, Beijing Normal–Hong Kong Baptist University, Zhuhai 519087, China

**Keywords:** porous convection, dynamic confinement, non-Darcy transition, Rayleigh–Bénard convections

## Abstract

Convection in porous materials governs heat transport across scales ranging from planetary subsurface systems to engineered cooling devices. While the onset of buoyancy-driven flow is well described by linear stability theory within a porous-continuum representation, the subsequent transition from viscous, matrix-dominated convection toward inertia-influenced and ultimately bulk fluid-like plume convection has lacked a unified description. Here we develop a confinement-based scaling framework that connects these flow states through a common scale-ratio perspective and quantitatively bridges classical porous convection with laterally confined Rayleigh–Bénard systems. Because random porous and fractured media do not admit an obvious static scale-ratio, we recover an effective confinement measure from the onset condition. This links permeability-based systems to the classical confinement framework and defines a characteristic pore length for natural convection. Comparing this pore length with the thermal boundary-layer thickness yields a dynamic criterion for the emergence of unconfined behavior. Embedding experimental and numerical porous–convection datasets into a unified phase diagram of buoyant forcing and static confinement reveals a systematic progression from viscous, drag-dominated heat transport to inertia-corrected flow and ultimately to plume-driven convection whose statistics approach those of unconfined fluids. The resulting framework delineates the limits of porous-continuum validity, clarifies when inertial corrections become relevant, and highlights the dynamical analogy between strongly confined porous flows and thin-gap Hele–Shaw configurations. By linking heat-transport scaling to static and dynamic length scales, the phase diagram provides a practical diagnostic for selecting appropriate governing equations across geophysical and engineered porous systems.

## Significance

Natural convection in porous and fractured materials governs heat transport in systems ranging from planetary hydrothermal circulation to engineered thermal technologies. Because the structure of these materials is often unknown, their flows are commonly described using continuum models that neglect pore geometry. We show that this approach breaks down when flow is strong enough: the structures controlling heat transport shrink below the pore size, so the solid framework no longer constrains the motion and the system behaves like convection in an unconfined fluid. By identifying this transition using length-scale ratios, we show when porous-media models remain valid and when pore-scale structure must be considered. This work clarifies the limits of modeling heat transport in porous materials when inertia becomes dominant.

Convection through porous and fractured materials is a fundamental mechanism governing heat transport in systems where a solid matrix is permeated by interconnected fluid-filled voids. Such systems span a broad range of geometries, porosities, and scales—from aquifers overlying magma chambers on planetary bodies (1, 2) to microscale cooling devices in electronic components (3). Identifying the governing dynamical regime is essential for selecting the appropriate equations and assessing the relative roles of buoyancy, drag, and inertia. This enables reliable first-order predictions of heat transfer and fluid motion, which are key for both scientific understanding and practical applications, from assessing hydrothermal circulation to designing efficient thermal-management systems (4).

In unconfined RB systems, convection arises from the competition between buoyant driving and the opposing effects of viscosity and thermal diffusion. This behavior is described by the incompressible, dimensionless Navier–Stokes–Fourier equations with Boussinesq-assumptions (5), where the Prandtl number Pr=ν/α characterizes the ratio of viscous to thermal diffusion, and the Rayleigh number $Ra=g\beta \Delta TH^{3}/\left(\nu \alpha \right)$ quantifies the strength of buoyant forcing.

Here, ν and α respectively denote the kinematic viscosity and thermal diffusivity; g, β, and ΔT respectively represent gravitational acceleration, thermal expansion, and the imposed temperature difference; and H is the system height. In this setting, the non-dimensional heat transport through the fluid layer, Nu_*f*_, and the control paramters Ra and Pr can be directly related to the globally averaged viscous and thermal dissipation rates, $\left\langle \varepsilon_{u}\right\rangle$ and $\left\langle \varepsilon_{T}\right\rangle$, respectively (6, 7). Using the standard Rayleigh–Bénard nondimensionalization based on length scale H, temperature scale ΔT, and the free-fall velocity scale $\sqrt{g\beta \Delta TH}$, the dissipation relations become

[1] $$\begin{array}{c} Nu_{f}\;=\;1+\left\langle \varepsilon_{u}\right\rangle {\left(Ra\;Pr\right)}^{1/2}\;=\;\left\langle \varepsilon_{T}\right\rangle {\left(Ra\;Pr\right)}^{1/2}, \end{array}$$

where ⟨·⟩ denotes a global space–time average, and subscripts u and T refer to velocity and temperature, respectively. The corresponding nondimensional dissipation rates are (8)

[2] $$\begin{array}{c} \begin{array}{cc} \varepsilon_{u} & ={\left(\frac{\mathrm{Ra}}{\mathrm{Pr}}\right)}^{-1/2}\sum_{i}\sum_{j}\frac{1}{2}(\frac{\partial u_{i}}{\partial x_{j}}+\frac{\partial u_{j}}{\partial x_{i}}{)}^{2},\\ \varepsilon_{T} & ={\left(Ra\;Pr\right)}^{-1/2}\sum_{i}{\left(\frac{\partial T}{\partial x_{i}}\right)}^{2}. \end{array} \end{array}$$

In porous media, the same fundamental competition between buoyant driving and viscous drag governs convection, but the presence of a solid matrix renders the problem conjugate, allowing heat conduction through both solid and fluid phases in contrast to fluid-only RB settings. In the absence of internal sources or sinks, the vertically integrated heat flux is conserved by Gauss’ theorem, so the Nusselt number can be recovered from the boundary fluxes as

[3] $$\begin{array}{c} Nu=Nu_{f}\;\frac{k_{f}}{k_{m}}. \end{array}$$

Here, $k_{f}$ is the fluid thermal conductivity and $k_{m}$ the effective conductivity used to define the purely conductive reference state of the fluid-saturated porous medium. Correspondingly, the kinetic dissipation relation in the fluid phase remains tied to the fluid Nusselt number, whereas the thermal dissipation expression assumes homogeneous diffusivity and in heterogeneous porous media requires the local thermal diffusivity to be introduced prior to averaging. While analogous instabilities may arise from solutal gradients (9, 10), thermal forcing can be sustained under fixed-temperature boundaries and permits steady heat transport. The present study therefore focuses on thermally driven convection, although many of the scaling considerations are expected to extend to solutal systems.

**Fig. 1.**
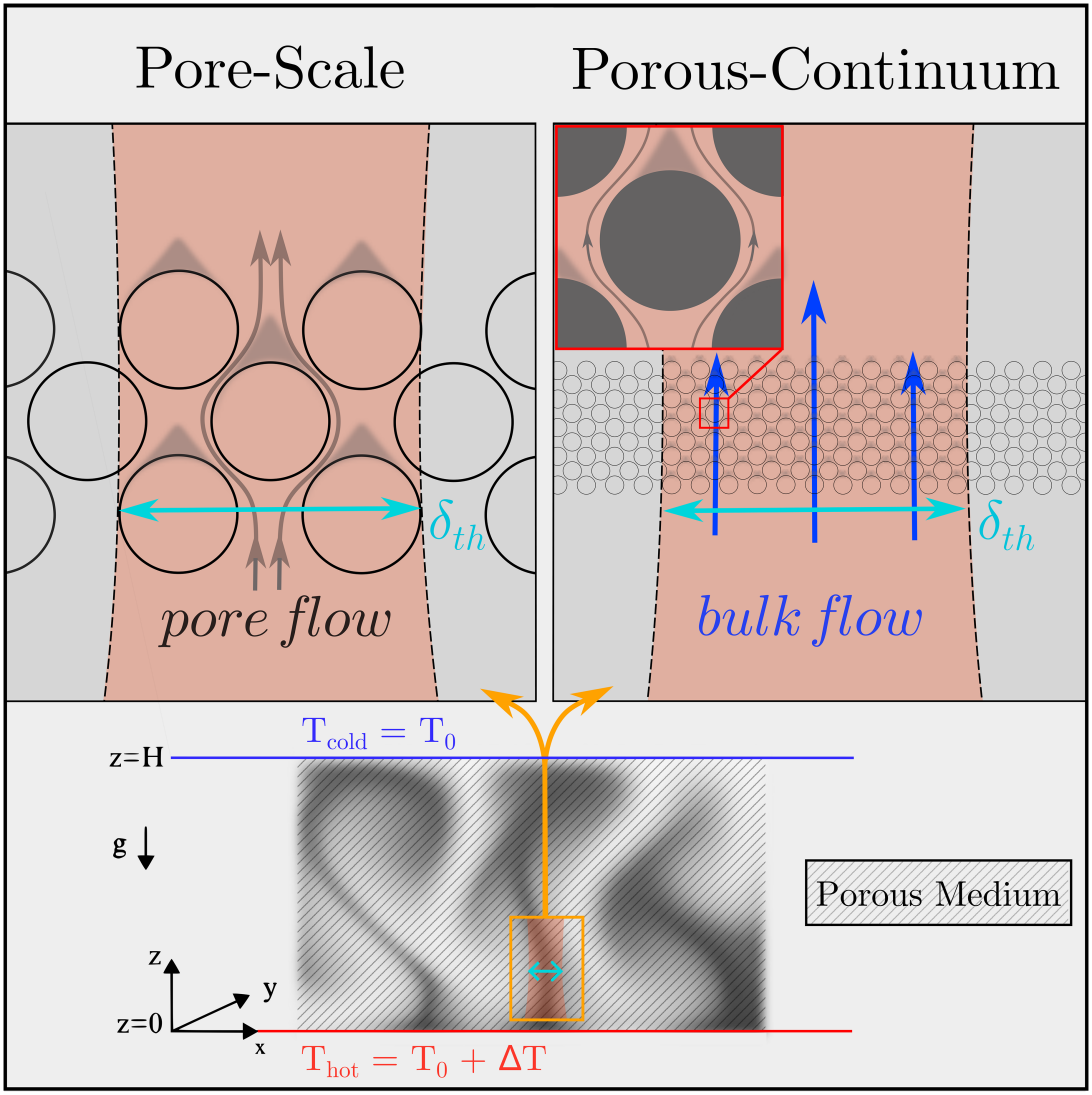
Pore-scale vs. porous-continuum perspectives of buoyancy-driven convection in porous media. *Left*: Dynamics confined to individual pores or smaller scales, where velocity and temperature fields are most appropriately resolved within a Navier–Stokes–Fourier framework and remain strongly dependent on the local pore geometry. *Right*: A mesoscopic view in which dominant thermal structures extend across multiple pores, indicating emerging scale separation and reduced pore-scale sensitivity, yet not constituting a fully valid porous-continuum limit. The thermal length scale at the plume neck, which is on the order of the thermal boundary-layer thickness, $\delta_{\mathrm{th}}$, serves as an indicator of whether the dynamics are governed primarily by pore-scale flow or by bulk throughflow across the porous layer.

In the absence of detailed geometric and boundary information needed for pore-resolved Navier–Stokes–Fourier simulations, convection in large porous domains is typically described using volume-averaged continuum formulations, including the Darcy–Oberbeck–Boussinesq (DOB) framework (11, 12). These continuum descriptions are commonly framed within the Horton–Rogers–Lapwood (HRL) problem (Fig. 1) and rely on scale separation between pore geometry and a representative elementary volume (REV) (13). In nondimensional form they are parameterized by effective quantities such as the Darcy number $Da=K/H^{2}$, with K being the porous medium permeability, effective thermal conductivity ratio $k_{f}/k_{m}$, porous drag coefficient $c_{F}$, and volumetric heat-capacity ratio (cf. equation 2.19 in ref. 12).

The onset of convection in an infinite horizontal porous layer bounded above and below by impermeable, isothermal surfaces occurs at a critical modified Darcy–Rayleigh number of $Ra_{c}^{*}=4\pi^{2}\approx 40$ (14). The corresponding modified Darcy–Rayleigh number is given by (14)

[4] $$\begin{array}{c} Ra^{*}=Ra\;Da\;\frac{k_{f}}{k_{m}}, \end{array}$$

which expresses the balance between buoyancy forcing and the dissipative effects of the porous matrix, including both momentum and thermal diffusion (15). Near onset, Darcy drag balances buoyancy, leading to steady convection in which heat transfer scales linearly as $Nu\propto Ra^{*}/Ra_{c}^{*}$ (16).

Wang and Bejan (17) analyzed the inertial regime by first considering an energy balance between vertical convective enthalpy transport and lateral thermal diffusion. To close this balance, they introduced a characteristic velocity scale obtained independently from a force balance between buoyancy and the empirical Forchheimer drag term describing inertial resistance in porous flow. Coupling these relations yields the porous-medium Prandtl number

[5] $$\begin{array}{c} Pr_{p}=\frac{\mathrm{Pr}}{c_{F}\sqrt{\mathrm{Da}}}\frac{k_{f}}{k_{m}}, \end{array}$$

which combines the relative roles of viscous, inertial, and thermal processes in inertia-dominated porous convection. Within this framework, Wang and Bejan argued that inertial effects become dominant when the Darcy–Rayleigh number exceeds the porous-medium Prandtl number ($Ra^{*}≳Pr_{p}$), marking the transition to a Forchheimer-type regime in which $Nu\propto {\left(Ra^{*}Pr_{p}\right)}^{1/2}$ (17). This criterion is supported by recent experimental observations (18, 19); however, several studies (20–22) exhibit departures from laminar scaling already at $Ra^{*}/Pr_{p}<1$ (23).

Laboratory measurements indicate that porous convection can progressively approach unconfined RB-like behavior under sufficiently strong thermal forcing in packed-bed configurations, with asymptotic heat-transport scalings such as $Nu=1.16{\left(Ra^{*}\right)}^{0.319}$ reported by Keene and Goldstein (24). Comparable effective exponents have also been reported in high-Rayleigh-number experiments employing sidewall heating to a porous layer (25) and in numerical studies of highly porous media (ϕ≳50%) (5, 12), with asymptotic behavior close to $Nu\propto {\left(Ra^{*}\right)}^{0.30}$. Expressed in terms of the corresponding fluid heat transport, the Keene and Goldstein scaling becomes $Nu_{f}=0.176\;Ra^{0.298}$. Despite the presence of a porous matrix in the former case, this scaling nearly coincides with the classical three-dimensional unconfined fluid relation $Nu_{f}=0.14\;Ra^{0.297}Pr^{-0.03}$ reported for Pr≈4 to 1,350 (26).

Overall, these empirically observed exponents in porous convection fall between the classical marginal-stability prediction $Nu\propto Ra^{1/3}$ and the $Nu\propto Ra^{2/7}$ scaling associated with mixing-zone and plume-based phenomenologies (27, 28), and are often slightly closer to 2/7. This indicates that once the flow approaches the asymptotic scaling branch, heat transport is not governed by an isolated marginally stable boundary layer, but instead reflects the coupled interaction between boundary layers and bulk dynamics (28). We note that a 2/7-type exponent has also been derived from shear-controlled turbulent boundary-layer arguments in high-Ra convection (6); however, that framework assumes a fully turbulent near-wall shear layer and a nested thermal boundary layer, conditions that are not evidently satisfied in the present confined porous configurations.

To explore the confined limit of RB convection, where lateral dimensions restrict plume growth and suppress large-scale circulation, Hele–Shaw (HS) cells serve as a canonical model (29, 30). Reducing the gap width b of a three-dimensional container decreases the aspect ratio Γ=b/H and progressively limits lateral plume development (8). In the thin-gap limit (b≪H), HS dynamics follow from a depth-averaged Navier–Stokes–Fourier reduction (30), in which the convective and temporal inertia terms are retained in averaged form. This contrasts with porous-media models, where inertial effects are commonly introduced through macroscopic closure relations such as Forchheimer drag rather than arising directly from the averaged momentum transport.

Building on RB convection observations in HS cells, Noto et al. (31) introduced the concept of plume-scale confinement, describing the transition from strongly confined to fully three-dimensional (3D) convection across the (Ra,Γ) parameter space. Confinement is quantified by the parameter $\Lambda =\delta_{\mathrm{th}}/b=\left(\delta_{\mathrm{th}}/H\right)\Gamma^{-1}$, where $\delta_{\mathrm{th}}$ denotes the thermal boundary-layer thickness controlling plume neck width. As Λ decreases from Λ>2 to Λ<1/20, they identify four regimes: i) a Darcy regime (Λ>2) characterized by inertia-free convection with Nu∝Ra, ii) a HS regime (1/2<Λ<2) in which the effective heat-transport exponent becomes Γ-dependent, $Nu\propto Ra^{\gamma (\Gamma )}$, iii) a partially 3D regime (1/20<Λ<1/2) where three-dimensionality first emerges near the boundaries, and iv) a fully 3D regime (Λ<1/20) whose statistics become indistinguishable from unbounded convection. The upper limit of the Darcy regime in HS cells is estimated from the inertia criterion ${\left(\Gamma^{2}/12\right)}^{2}Ra\ll 1$ (practically $<10^{-2}$) (30), yielding the Darcy boundary $\Gamma_{D}=\sqrt{6/5}\;Ra^{-1/4}$. To evaluate Λ, they employ the RB-type asymptotic heat-transport scaling of Xia et al. (26), introduced above.

Experimental evidence (19) and theoretical considerations (17) in HRL convection indicate that porous-continuum-like dynamics can persist even when inertia becomes significant. This implies that static confinement and inertial effects are not mutually exclusive: the flow may remain kinematically confined (Λ>2) while dynamically departing from Darcy behavior (8, 23). Moreover, a quantitative, dynamic criterion linking geometry and forcing to the appropriate governing framework remains missing for porous convection, i.e., whether heat transport is captured by a volume-averaged porous-medium description (Darcy/Forchheimer) or instead requires a Navier–Stokes–Fourier treatment (RB-type).

In this study, we retain the classical RB length-scale ratio Γ=b/H and introduce a porous-medium translation that is linked to the Darcy number and constrained by the onset of convection. This static confinement measure places porous-medium and RB convection within a common aspect-ratio framework and establishes a direct conceptual bridge between the HRL convection and classical RB problems. Using this framework, we revisit the relations of Wang & Bejan (equations 18 and 19 in ref. 17), clarify their range of validity, and evaluate them against a broad compilation of experimental and numerical porous–convection datasets. We further embed these data into a common phase diagram originally formulated for confined RB convection (8, 31), extending this representation to porous-media convection ($\Gamma <10^{-3}$). The present synthesis focuses on intermediate to large Darcy numbers ($Da∼10^{-4}$ to $10^{-8}$), corresponding to the transition from porous-continuum to pore-scale flow, where the compiled data show increasing similarity to classical unconfined convection behavior.

## Results and Discussion

To enable a direct dynamic comparison between laterally confined RB convection and the HRL convection in porous media, it requires a geometrical mapping between the lateral confinement scale and an effective pore length scale. Such a mapping is physically meaningful because of their shared onset behavior between the two types of convection: the critical instability in strongly confined RB configurations (e.g., HS cells) and in classical porous layers converges to a common critical value when expressed in terms of the modified Darcy–Rayleigh number ($Ra_{c}^{*}∼4\pi^{2}$) (14, 32). Consistent with this observation, both the finite–aspect–ratio analysis of Shishkina et al. (33) and the numerical results of Chong et al. (8) in strongly confined HS cells demonstrate a systematic shift of the critical Rayleigh number with lateral confinement Γ, which can be approximated as (31, 33)

[6] $$\begin{array}{c} Ra_{c}\approx {\left(2\pi \right)}^{4}\left(1+\frac{1}{4\Gamma^{2}}\right), \end{array}$$

consistent with linear stability analysis for finite–aspect–ratio cells and reflecting the asymptotic $Ra_{c}\propto \Gamma^{-2}$ increase under strong lateral confinement.

Taking reported values of $Ra_{c}$, the effective pore length scale b in the static confinement Γ=b/H can be derived as

[7] $$\begin{array}{c} b\approx C\;\sqrt{\frac{K}{Ra_{c}^{*}}\;\frac{k_{f}}{k_{m}}}. \end{array}$$

Here $\sqrt{K}$ represents an experimentally accessible equivalent pore opening, whereas $\sqrt{k_{f}/k_{m}}$ typically provides only a secondary correction relative to the orders-of-magnitude variation of Da. For the commonly observed onset $Ra_{c}^{*}\approx 4\pi^{2}$, the prefactor C in Eq. **7** reduces to C≈π, recovering the purely static confinement in HS: $\Gamma ∼\pi \sqrt{\mathrm{Da}}$ for $k_{f}=k_{m}$ (details of the derivation are provided in *Materials and Methods*). Heterogeneities in permeability and thermal conductivity of porous media can influence the precise critical value of b (34); this effect is explicitly accounted for where reported (18) or obtained from literature otherwise (22). Most laboratory and numerical configurations considered here employ domain aspect ratios close to unity and the resulting shifts remain within the same order of magnitude.

**Fig. 2.**
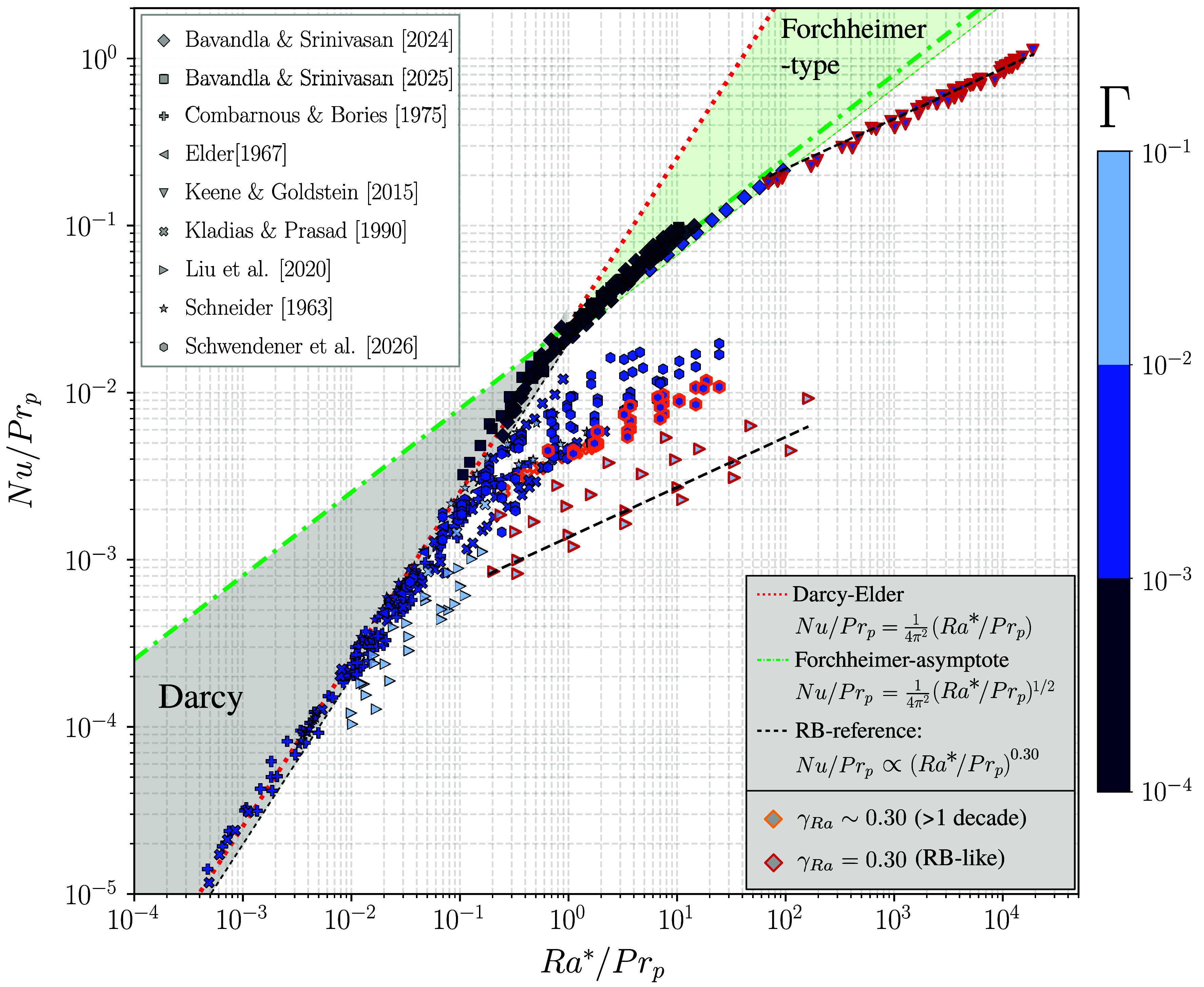
Scaling of the Nusselt number Nu vs. the modified Darcy–Rayleigh number $Ra^{*}$ for compiled experimental and numerical HRL convection datasets, where both Nu and $Ra^{*}$ are scaled by the porous-medium Prandtl number $Pr_{p}$ as $Nu/Pr_{p}$ and $Ra^{*}/Pr_{p}$, respectively. Data points represent compiled experimental and numerical studies (Tables 1–3). Colors denote the static confinement parameter Γ. The red dotted line indicates the Darcy–Elder scaling $Nu/Pr_{p}={\left(4\pi^{2}\right)}^{-1}\left(Ra^{*}/Pr_{p}\right)$, while the green dash-dotted line shows the Forchheimer-asymptote $Nu/Pr_{p}={\left(4\pi^{2}\right)}^{-1}{\left(Ra^{*}/Pr_{p}\right)}^{1/2}$. Shaded regions provide visual guidance for Darcy-dominated behavior and proximity to the Forchheimer-asymptote, while data points exhibiting sustained fluid-like scaling ($\gamma_{\mathrm{Ra}}∼0.30$ over approximately one decade in Ra) are highlighted by orange rims, and cases that additionally align with the asymptotic reference fluid scaling are marked by red rims (5, 24).

Reexamining the laminar–inertial transition criterion $Ra^{*}∼Pr_{p}$ proposed by Wang and Bejan (17) reveals a systematic dependence on static confinement (Fig. 2). The theoretical Darcy–Elder and Forchheimer asymptotes are included to illustrate the corresponding heat-transport scalings arising from empirical force balances between buoyancy and Darcy or Forchheimer drag within the HRL framework. For orientation, the compiled datasets are grouped into Darcy–Elder, Forchheimer-asymptotic, transitional, and asymptotic RB-type behaviors, with the lower boundary between Darcy and Forchheimer ranges inferred from a combined uncertainty estimate of the reported Rayleigh, Darcy, and Nusselt values (see *Materials and Methods* for details). Cases are marked with orange rims when the data are compatible with $Nu\propto Ra^{\gamma_{\mathrm{Ra}}}$ with $\gamma_{\mathrm{Ra}}\approx 0.30$ over approximately one decade in Ra, and with red rims when they follow the reference fluid scaling, indicating asymptotic behavior (5, 24).

**Fig. 3.**
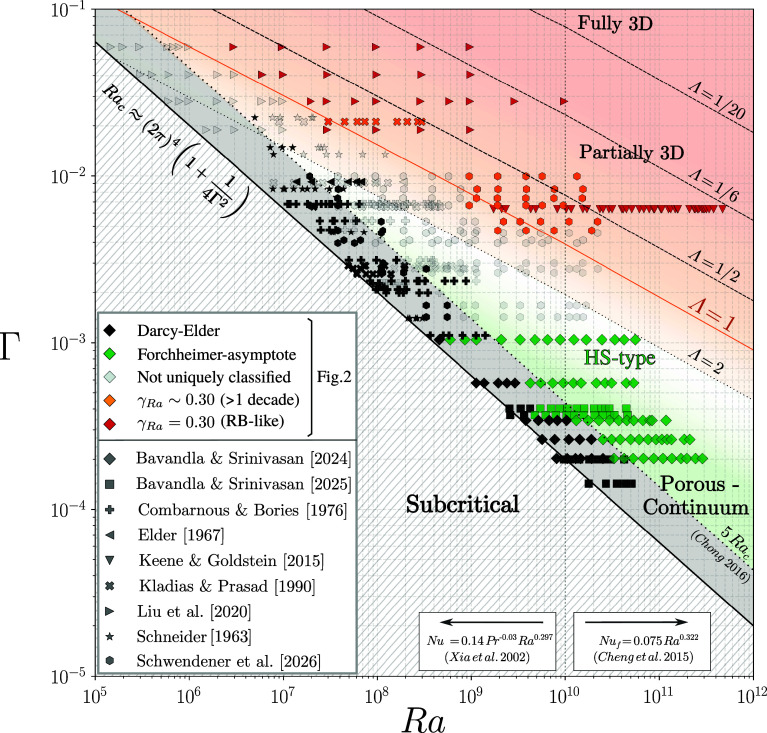
Phase diagram of HRL convection in the (Ra,Γ) domain constructed from the datasets shown in Fig. 2. Data points correspond to those compiled in Fig. 2 and are grouped into five categories: i) black, following Darcy–Elder scaling; ii) green, approaching the Forchheimer asymptote; iii) orange, compatible with $\gamma_{\mathrm{Ra}}∼0.30$ over at least one decade in Ra; iv) red, aligning with the asymptotic reference fluid scaling (5, 24, 26); and v) unclassified, representing transitional cases that follow neither Darcy–Elder nor the Forchheimer asymptote and do not exhibit sustained asymptotic scaling. The black solid line marks the confinement-dependent onset criterion described in Eq. **6**. Dashed isolines of the dynamic confinement parameter $\Lambda =\delta_{\text{th}}/b$ are estimated from unconfined heat-transport correlations using $\delta_{\text{th}}\approx H/\left(2Nu\right)$ (26, 38) at representative Pr=1, indicating where the evolving thermal boundary-layer thickness becomes comparable to the effective pore length scale b (Eq. **7**). Background shading provides visual guidance for dynamical similarity: strongly confined behavior near onset ($Ra\leq 5Ra_{c}$), intermediate HS-type dynamics for Λ∼O(1), and progressively three-dimensional pore-scale dynamics as Λ decreases below unity, where characteristic length scale of flow structures becomes smaller than the pore spacing. In thin-gap HS cells, comparable dynamical transitions are typically observed at slightly smaller Λ∼1/2, as discussed in the text.

The theoretical transition criterion is largely supported by the recent experimental datasets of Bavandla and Srinivasan (18, 19), for which the transition occurs under strong static confinement ($\Gamma ≲10^{-3}$) and the data approach the Forchheimer-asymptote. As static confinement is progressively relaxed, several datasets (5, 15, 16, 23) depart early from the classical Darcy–Elder scaling. At larger static aspect ratios ($\Gamma ≳10^{-2}$), the data show increasing deviations from this linear, onset–type heat–transport behavior and trend toward shallower intermediate scaling, approaching $\gamma_{\mathrm{Ra}}∼0.65$ (5), before transitioning toward the classical RB scaling. In this intermediate scaling range, the thermal fields exhibit organized large-scale circulation, typically consisting of domain–spanning circulation with laterally repeating rolls (e.g., figure 7G in ref. 5; figures 3 and 5 in ref. 23). Despite locally elevated pore-scale velocities sufficient to generate significant inertial effects, the global flow field typically remains steady or exhibits only weak pore-scale harmonic oscillations, indicating partial stabilization by the porous matrix (5, 23). Although the plume neck remains larger than the characteristic pore opening, the dynamics approach this geometric limit and become effectively comparable to HS configurations, resembling pore-scale confinement (Fig. 1). A related energetic balance is observed in thermally driven HS convection, where kinetic-energy decay is governed by a combination of Darcy-like friction and Stokes viscous dissipation (35, 36). The corresponding thermal fields, however, exhibit stronger lateral plume spreading due to the absence of porous-matrix stabilization (figure 5 in ref. 8).

Within the porous, Prandtl-corrected $Ra^{*}$–Nu framework, Wang and Bejan (17) proposed a Churchill–Usagi-type power law (37) to unify laminar and inertial regimes. When reexamined under the present confinement formulation, however, the transition is found to depend strongly on Γ, such that the reported coefficients (see equation 19 in ref. 17) effectively average over a broad intermediate transition rather than representing a distinct scaling limit. We therefore do not introduce such Churchill–Usagi-type equation, as the Forchheimer relation is more appropriately interpreted as an intermediate state than as an indefinitely asymptotic regime.

For a complementary perspective, the sensitivity of Γ is recast in terms of a dynamic confinement measure, Λ, which relates the evolving thermal boundary layer to the pore geometry. The HRL convection datasets (Fig. 2) are mapped onto the (Ra,Pr,Γ) phase space (Fig. 3), enabling direct comparison between porous (i.e., HRL), thin-gap (e.g., HS), and RB configurations (8, 31). Isolines of Λ follow the relationship $\Gamma =1/(2\;Nu_{f}\;\Lambda )$ with $\delta_{\mathrm{th}}\approx H/\left(2Nu_{f}\right)$ (31), where $Nu_{f}$ is estimated from unconfined correlations, $Nu_{f}\propto Ra^{0.297}Pr^{-0.03}$ for $Ra≲10^{10}$ (26) and $Nu_{f}\propto Ra^{0.322}$ at higher Ra (38). Across the compiled datasets, the Prandtl number is strongly concentrated in the range Pr≈0.7 to 7, with only a small number of intermediate cases around Pr≈15 to 25 and two high-Pr datasets near $Pr∼10^{2}$ (Tables 1–3). Given the weak Pr dependence of unconfined heat transport, the phase space is evaluated at a representative Pr=1, with only minor variation expected in the estimated isolines (Λ≤2). The line $5\;Ra_{c}$ is included to indicate the onset range for laterally confined convection (8, 14).

**Table 1.** Recent laboratory HRL experiments (2015–2025)

Dataset	Fluid	Solid	Pr	$k_{m}/k_{f}$	$Da\;[\times 10^{-6}]$	$Ra_{c}^{*}$
S1 (18)	Ar	Gl	0.7	17.61	1.94	$4\pi^{2}$
S2 (18)	Ar	Gl	0.7	15.68	0.52	$4\pi^{2}$
S3 (18)	Ar	Gl	0.7	18.47	0.24	$4.34\pi^{2}$
S4 (18)	Ar	Gl	0.7	18.13	0.16	$5.14\pi^{2}$
S5 (18)	Ar	Gl	0.7	18.58	0.12	$6.25\pi^{2}$
S6 (19)	Ar	Al	0.7	34.28	0.47	$4\pi^{2}$
S7 (19)	Ar	Al	0.7	27.51	0.06	$4\pi^{2}$
S8 (19)	Ar	Gl	0.7	14.6	0.06	$4\pi^{2}$
S9 (19)	Ar	St	0.7	29.16	0.48	$4\pi^{2}$
S20 (24)	Ar	PP	0.7	5.04	21.8	$4\pi^{2}$

Abbreviations: Ar = argon; Gl = glass, St = steel, Al = aluminum, PP = polypropylene.

**Table 2.** Historic HRL datasets

Dataset	Fluid	Solid	Pr	$k_{m}/k_{f}$	$Da\;[\times 10^{-6}]$	$Ra_{c}^{*}$
S10 (15)	Oil	Gl	123.1	1.7	1.0	$4\;\pi^{2}$
S11 (15)	Oil	Gl	123.1	1.7	0.2	$4\;\pi^{2}$
S12 (15)	W	Gl	4.8	1.0	4.8	$4\;\pi^{2}$
S13 (15)	W	Gl	4.8	1.0	3.0	$4\;\pi^{2}$
S14 (15)	W	Gl	4.8	1.0	1.0	$4\;\pi^{2}$
S15 (15)	W	Pb	4.8	2.1	4.8	$4\;\pi^{2}$
S16 (15)	Oil	Qz. S.	123.1	2.1	0.9	$4\;\pi^{2}$
S17 (15)	W	Qz. S.	4.8	1.9	1.0	$4\;\pi^{2}$
S18 (15)	W	Qz. S.	4.8	1.9	0.9	$4\;\pi^{2}$
S19 (16)	W	Gl	6.9	1.2	9.9	$4\;\pi^{2}$
S21 (22)	W	Acr	5.7	0.6	25.8	$4\;\pi^{2}$
S22 (22)	W	Gl	5.5	1.4	1.2	$4\;\pi^{2}$
S23 (22)	W	Gl	5.4	1.4	5.8	$4\;\pi^{2}$
S24 (22)	Gly	Gl	112.8	2.3	1.5	$4\;\pi^{2}$
S25 (22)	W	St	5.9	5.9	68.5	55
S26 (22)	W	St	5.6	7.7	6.0	$4\;\pi^{2}$
S31 (20)	Trp	Gl	20.9	3.1	57.5	$4\;\pi^{2}$
S32 (20)	Trp	Gl	20.9	3.1	0.6	$4\;\pi^{2}$
S33 (20)	W	Gl	6.2	1.2	61.9	$4\;\pi^{2}$
S34 (20)	W	Gl	6.2	1.2	27.2	$4\;\pi^{2}$
S35 (20)	W	Gl	6.2	1.2	8.6	$4\;\pi^{2}$
S36 (20)	W	Gl	6.2	1.2	2.6	$4\;\pi^{2}$
S37 (20)	W	Gl	6.2	1.2	0.6	$4\;\pi^{2}$
S38 (20)	Trp	St	20.9	15.6	11.1	$4\;\pi^{2}$

Abbreviations: W = water, Gly = glycol, Trp = turpentine; Gl = glass, Acr = acrylic, St = steel, Pb = lead, Qz. S. = quartz sand.

**Table 3.** Numerical HRL datasets

Dataset	Type	Pr	$k_{m}/k_{f}$	$Da\;[\times 10^{-6}]$	$Ra_{c}\;\left[\times 10^{6}\right]$
S27 (5)	FDM	4.3	1.00	1300	0.112
S28 (5)	FDM	4.3	1.00	450	0.238
S29 (5)	FDM	4.3	1.00	180	0.498
S30 (5)	FDM	4.3	1.00	75	1.09
S39 (23)	LBM	1.0	3.77	0.905	192.0
S40 (23)	LBM	1.0	2.73	0.905	139.0
S41 (23)	LBM	1.0	1.00	0.905	50.8
S42 (23)	LBM	1.0	0.37	0.905	18.8
S43 (23)	LBM	1.0	0.27	0.905	13.7
S44 (23)	LBM	1.0	3.22	2.62	56.5
S45 (23)	LBM	1.0	2.46	2.62	43.2
S46 (23)	LBM	1.0	1.00	2.62	17.6
S47 (23)	LBM	1.0	0.41	2.62	7.20
S48 (23)	LBM	1.0	0.32	2.62	5.62
S49 (23)	LBM	1.0	2.84	5.25	24.9
S50 (23)	LBM	1.0	2.62	5.25	23.0
S51 (23)	LBM	1.0	1.00	5.25	8.76
S52 (23)	LBM	1.0	0.45	5.25	3.94

Abbreviations: FDM = finite difference method; LBM = Lattice Boltzmann method. Onset values are fluid Rayleigh numbers scaled as $Ra_{c}\;\left[\times 10^{-6}\right]$.

The transition to quasi-3D convection in HS cells, marked by the loss of lateral confinement and a shift toward the classical unconfined heat–transport behavior, has been reported to occur at slightly lower values of Λ≲0.5 than those observed here for convection in porous media, where a comparable fluid-like state emerges near Λ∼1.0 [Fig. 3; datasets of Liu et al. (5) and Keene & Goldstein (24)]. From the present perspective, two aspects are particularly relevant for this comparison. First, two-dimensional (2D) porous-media studies are often formulated with periodic boundary conditions in the out-of-plane (suppressed) dimension, which may reduce the effective drag acting on the flow relative to thin-gap HS configurations (5, 8, 23). Second, porous media combine pore throats and pore bodies, so local confinement at the throat coexists with a connected network that offers multiple pathways for redistribution and plume self-organization. In contrast, a thin-gap HS cell enforces motion within a single gap-defined plane, representing a geometric extreme of lateral restriction that limits such reconfiguration (31). This distinction is consistent with 3D visualizations of porous convection, which demonstrate that the flow can organize in multiple 3D patterns (39, 40); HS cells therefore approximate the limiting case of porous convection confined to a single fracture of smooth and straight surfaces.

The datasets of Schwendener et al. (23) lie predominantly within the transitional band between Darcy–Elder and fluid-like convection, spanning both a dynamical transition from laminar, Darcy-dominated to inertially influenced flow and a geometric transition from confined to effectively unconfined convection. Within that study, reducing Γ at comparable Ra induced a systematic steepening of the $Nu\propto Ra^{\gamma_{\mathrm{Ra}}}$ scaling from $\gamma_{\mathrm{Ra}}\approx 0.30$ toward an intermediate Forchheimer–type behavior ($\gamma_{\mathrm{Ra}}\approx 0.5$). This trend continues for $\Gamma ≲10^{-3}$ and $Ra>5\;Ra_{c}^{*}$, whereas the datasets of Bavandla and Srinivasan (18, 19) align with the theoretical Forchheimer-asymptote (Fig. 2). Consistently, a systematic increase in $\gamma_{\mathrm{Ra}}$ with decreasing Γ is observed, as evident from figure 7 of ref. 18. Dynamically, this confinement state closely resembles strongly confined HS experiments, where $\gamma_{\mathrm{Ra}}$ likewise varies with Γ (31) and intermediate behavior has been observed to extend beyond Λ≳2 (see figure 2 in ref. 8). This trend is consistent with increased geometrically imposed viscous drag under strong lateral confinement, as also reflected in Reynolds-number analyses (8, 23), where the velocity scaling is observed to steepen systematically with decreasing Γ.

The transition toward nonlaminar convection in porous media cannot be sharply resolved within the fluid (Γ,Ra) framework alone. For strong confinement ($\Gamma ∼O(10^{-4})$), the compiled data remain consistent within the porous-continuum interpretation, and $Ra^{*}/Pr_{p}$ can serve as a practical indicator of inertial effects (Fig. 2). Formally, porous–continuum models require large dynamic confinement (Λ≫1) to ensure scale separation between the pore structure and the thermal boundary layer (13). Consistent with this requirement, the datasets of Bavandla and Srinivasan correspond to Λ≳O(10) and show a transition toward nonlaminar convection at $Ra^{*}/Pr_{p}∼1$ (23).

An alternative approach invokes a pore-scale Reynolds number as a transition criterion (14, 19). However, $Re_{p}$ is not an independent control parameter but depends on the chosen characteristic velocity and pore length scale. With a buoyancy–Forchheimer balance, $Ra^{*}/Pr_{p}=c_{F}^{2}Re_{p}^{2}$ (19) and since $Ra^{*}/Pr_{p}\propto Ra\;\Gamma^{3}$, a constant Reynolds threshold corresponds to $Ra\propto \Gamma^{-3}$ in the (Ra,Γ) plane. Using instead the inertial (free-fall) estimate $U∼\sqrt{g\beta \Delta TH}$ gives $Re_{p}∼\Gamma \sqrt{Ra/Pr}$, so that a constant $Re_{p}$ implies $Ra\propto \Gamma^{-2}$, corresponding to lines parallel to $Ra_{c}\left(\Gamma \right)$ in Fig. 2. These alternative scalings indicate that the progression from laminar to unconfined convection is gradual rather than sharply defined; inertial effects develop progressively once Ra exceeds a few multiples of $Ra_{c}\left(\Gamma \right)$, consistent with confined HS simulations in ref. 8.

To translate the above considerations into practical parameter ranges, the scaling of the Darcy–non-Darcy criterion can be examined more explicitly. Eqs. **4** and **5** yield $Ra^{*}/Pr_{p}=c_{F}\;g\;\beta \;\Delta T\;\sqrt{K^{3}}/\nu^{2}$, suggesting that this ratio is governed primarily by permeability K and kinematic viscosity ν, whereas the remaining parameters typically vary only within order–unity bounds for geological systems. Typical geofluids commonly encountered in the Earth’s crust (e.g., in the vicinity of magmatic intrusions), adopting $c_{F}\;g\;\beta \;\Delta T∼O\left(1\right)$(41) reduces the scaling to $Ra^{*}/Pr_{p}\propto \sqrt{K^{3}}/\nu^{2}$. For representative kinematic viscosities of hot, pressurized H_2_O-rich fluids, including near-critical and supercritical water (1), $\nu ∼10^{-7}\;m^{2}\;s^{-1}$, the transition region is therefore expected to emerge only at comparatively large permeabilities, $K≳10^{-9}\;m^{2}$. Such values exceed those typically reported for intact natural rocks, for which permeabilities are often $K\leq 10^{-14}\;m^{2}$ (1), thereby justifying the small-Darcy-number assumption ($Ra^{*}/Pr_{p}\ll 1$) commonly adopted in finite-Darcy-limit studies (11, 42). Open fractures can increase effective permeability of formations by several orders of magnitude, with reported values for volcanic rocks at laboratory-scale around $K∼10^{-12}\;m^{2}$ (43). Because K enters the scaling with exponent 3/2, increases in permeability or reductions in viscosity (e.g., supercritical fluids or gases) can shift the system away from strictly laminar behavior. Nevertheless, under porous or weakly fractured crustal conditions with aqueous fluids, laminar Darcy-type convection remains the prevailing expectation (2).

In engineered porous systems, such as packed beds in high-temperature gas-cooled reactors or open-cell foams used for thermal management (3, 25, 44–46), the attainable permeabilities are commonly much higher than those of geological formations, so departures from the laminar Darcy limit are expected and dynamical behavior can be readily estimated from the Γ−Ra phase diagram.

To provide a practical first-order orientation for assessing porous convection across applications, the following parameter-based indicators may be considered:

- **Estimate static confinement Γ** either from direct measurements (e.g., permeability K and effective thermal conductivity $k_{m}$, Eq. **7**) or from reported onset values $Ra_{c}$, and position the operating range within the Γ–Ra phase diagram shown in Fig. 3. Empirical correlations may be used where direct measurements are unavailable (14).
- **Confinement range investigated here.** Within the parameter range covered in the present study, pore-scale resolution is required and direct Navier–Stokes–Fourier simulations (23) are necessary to distinguish laminar, inertial, or turbulent dynamics.
- **Substantially lower confinement.** For confinement levels markedly smaller than those considered here ($\Gamma \ll 10^{-4}$; e.g., unfractured natural rocks with $k≲10^{-14}\;m^{2}$), a Darcy-type porous–continuum description is generally expected to remain adequate. In this limit, estimation of both $Pr_{p}$ and $Ra^{*}$, followed by comparison of their magnitudes, offers an initial assessment of whether the convection is in the Darcy regime; specifically, $Ra^{*}\ll Pr_{p}$ signals Darcy–dominated dynamics. If $Ra^{*}\ll Pr_{p}$ is not satisfied, inertial (Forchheimer) or, at very high porosity, Brinkman and dispersion corrections may become relevant (14).
- **Dynamic confinement check.** The dynamic confinement Λ serves as a validity indicator: values relaxing downward toward Λ∼O(10) imply diminishing scale separation and increasing uncertainty of porous-continuum assumptions (23). In contrast, Λ≪1, together with the low Mach-number condition, outlines the upper dynamical limit of the incompressible Navier–Stokes–Fourier framework and indicates the spatial resolution required to adequately resolve the relevant dynamics.

Future work aimed at understanding porous convection across laboratory to field scales should extend experimental constraints to substantially lower static confinements, in conjunction with geophysical evidence (47), to more rigorously test the empiric Darcy-continuum framework and improve calibration of empirical closure terms (48, 49). An alternative and promising route lies in systematic upscaling of the governing Navier–Stokes–Fourier equations (50); however, consistent coupling of the resulting macroscopic relations and estimation of the associated kernel functions remain open challenges. On the numerical side, resolving low-permeability regimes at high resolution and extending simulations to three dimensions remain computationally demanding but are essential for validating convection patterns observed in experiments (19, 40).

## Conclusion

We establish a confinement-based (Ra,Γ) scaling framework for porous/fractured systems by constraining the dynamics at the onset of convection, thereby providing a quantitative bridge between the classical Horton–Rogers–Lapwood (HRL) and Rayleigh–Bénard (RB) convection problems. Revisiting the canonical transition criterion $Ra^{*}∼Pr_{p}$, we show that it remains valid only under strong static confinement ($\Gamma ≲10^{-3}$). When calibrated across mixed confinement levels, Churchill–Usagi-type correlations yield fitting coefficients that become misleading when applied beyond their confined calibration range, because the implicit Γ dependence is unresolved (17). Dynamically, convection with Forchheimer-type corrections exhibits close similarity to strongly confined Hele–Shaw (HS) behavior and is more appropriately interpreted as a transitional state rather than a distinct asymptotic regime. An expansion of this intermediate behavior toward lower Γ may be anticipated from the (Ra,Γ) phase diagram. Unconfined convection with RB reference scaling ($\gamma_{\mathrm{Ra}}\approx 0.30$) emerges for Λ≲1, slightly earlier than the reported transition from HS results, which we attribute here to the additional degrees of freedom inherent to porous media, compared to the restricted HS plane. In practical terms, the transition to inertial HRL convection is governed predominantly by permeability and kinematic viscosity: under typical crustal geofluid conditions, the laminar Darcy limit remains the prevailing expectation, whereas elevated permeabilities or reduced viscosities, as encountered in engineered porous systems, shift the system toward inertial or bulk-fluid behavior.

## Materials and Methods

### Effective Pore Length Scale from Onset Criterion.

Starting from the finite–aspect–ratio approximation $Ra_{c}\approx {\left(2\pi \right)}^{4}\left(1+\frac{1}{4\Gamma^{2}}\right)$, the strong-confinement limit (Γ≪1) yields $Ra_{c}\approx 4\pi^{4}/\Gamma^{2}$ and hence $\Gamma \approx 2\pi^{2}/\sqrt{Ra_{c}}$. Expressing the fluid Rayleigh number through the commonly reported Darcy–Elder onset $Ra_{c}^{*}$ gives $Ra_{c}=Ra_{c}^{*}/\left(Da\;k_{f}/k_{m}\right)$, so that

$$\Gamma \approx \frac{2\pi^{2}}{\sqrt{Ra_{c}^{*}}}\sqrt{Da\;\frac{k_{f}}{k_{m}}}.$$

With $Da=K/H^{2}$ and Γ=b/H this yields

$$b\approx \frac{2\pi^{2}}{\sqrt{Ra_{c}^{*}}}\sqrt{K\;\frac{k_{f}}{k_{m}}}.$$

For the frequently observed $Ra_{c}^{*}\approx 4\pi^{2}$, the prefactor reduces to π, recovering $b\approx \pi \sqrt{K\;k_{f}/k_{m}}$.

### Supporting Datasets.

Datasets with limited coverage of the onset region were generally excluded (22), except for the high-Rayleigh-number measurements of Keene and Goldstein (24). The treatment of $Ra_{c}$ is discussed below. Studies with very small height-to-bead ratios, for which the porous drag coefficient $c_{F}$ cannot be estimated reliably (Eq. **9**) (20, 22, 51, 52), as well as experiments conducted in high-porosity foams (44, 53, 54), predominantly fall in the high-Γ regime ($\Gamma ≳10^{-2}$), which is represented here by the pore-resolved study of Liu et al. (5).

The dataset of Buretta and Bermann (21) was not considered because variations in layer height modify the Darcy number across cases, while the corresponding changes in Da are difficult to resolve explicitly, preventing consistent integration into the present analysis. Nevertheless, the data illustrate the same overall convective progression discussed here (see Layers A–D in figures 2 and 3 of ref. 21). The classic experiments of Elder (16), while illustrative, often lack sufficient auxiliary parameters for quantitative comparison.

The direct numerical simulations of Korba et al. (12) for fully saturated square arrays, as well as the three-dimensional simulations of ref. 52, are valuable complementary studies of the weak-confinement regime, which is represented here by the study of Liu et al. (5). Solutal (density-driven) convection studies (55) display analogous dynamical trends but are beyond the scope of the present work, which is restricted to thermally driven buoyancy.

### Parameter Estimation: K, $c_{F}$, $Pr_{p}$, $Ra_{c}$.

For experimental studies in packed–bed geometries that do not report the Darcy number (15, 16, 20), the permeability K was estimated using the Carman–Kozeny relation with a dimensionless shape factor of 180 for random packed beds (19),

[8] $$\begin{array}{c} K=\frac{d^{2}\varphi^{3}}{180\;{\left(1-\varphi \right)}^{2}}, \end{array}$$

where d denotes the characteristic grain diameter and φ the porosity.

To estimate the inertial drag coefficient required for computing $Pr_{p}$, we employed the correlation of Nield and Bejan (14),

[9] $$\begin{array}{c} c_{F}=0.55\left(1-5.5\;\frac{d}{D_{e}}\right),\;D_{e}=\frac{2LH}{L+H}, \end{array}$$

where L and H are the horizontal and vertical cavity dimensions. Their harmonic mean $D_{e}$ represents an effective hydraulic length scale that accounts for geometric confinement in both directions. Datasets for which this relation yields negative values of $c_{F}$ (indicating excessive static confinement and violation of the porous–continuum assumption) were excluded (22, 23).

For the datasets of Liu et al. [S27–S30 (5)], the porous drag coefficient was taken from the tabulated values reported by Khalifa et al. (56) (denoted α in their Table 3 for porosities between 0.7 and 0.9), thereby maintaining consistency with the original experimental conditions. Although alternative estimators for $c_{F}$ exist (14, 19), they likewise rely on the validity of a porous-continuum description and are therefore not robust under large static confinement.

Porous Prandtl numbers were adopted from refs. (19, 23), except for the datasets S21–S26 (22), S20 (24) and S27 to S30 (5), for which $Pr_{p}$ was calculated consistently using the expressions above (values in *SI Appendix*, Tables S1–S3).

The critical Rayleigh numbers, $Ra_{c}$, were adopted from reported theoretical considerations for Datasets S1–S9 (18, 19), whereas for Datasets S10–S26 (15, 16, 22) and S31–S38 (20) they were taken from experimental observations. The associated uncertainty is conservatively estimated to be on the order of ±10% (16, 18); because the inferred confinement scales as $\Gamma \propto Ra_{c}^{-2}$ and all cases considered satisfy Ra≫1, this level of uncertainty does not affect the qualitative classification in the confined limit (Tables 1 and 2). The dataset of Keene and Goldstein [Dataset S20 (24)] is the only large experimental study that does not explicitly report onset behavior; therefore, the critical modified Darcy–Rayleigh number was assumed as $4\pi^{2}$. This represents a conservative estimate, as their configuration employs a square domain and boundary conditions comparable to other laboratory experiments (19, 24). For the numerical studies [Datasets S27–S30 (5) and S39–S52 (23)], the critical Rayleigh number $Ra_{c}$ was estimated directly from the lowest available onset points using a linear extrapolation of Nu in the near-onset regime. This procedure provides a consistent first-order approximation of the instability threshold and the resulting values are summarized in Table 3.

To indicate the uncertainty of the theoretical Darcy and Forchheimer bounds in Fig. 2, we propagate representative relative errors (16, 19, 24) in the abscissa $x=Ra^{*}/Pr_{p}$ and ordinate $y=Nu/Pr_{p}$ into multiplicative bands around the Darcy (Elder) and Forchheimer asymptotes. Assuming independent uncertainties of 5% in $Ra_{f}$ and $k_{m}/k_{f}$, a 15% “Darcy-type” uncertainty entering x and y as $Da^{3/2}$ and $Da^{1/2}$, and 5% in Nu, we obtain $\sigma_{\ln x}=\sqrt{\sigma_{Ra_{f}}^{2}+\sigma_{k}^{2}+{\left(1.5\;\sigma_{\mathrm{Da}}\right)}^{2}}$ and $\sigma_{\ln y}=\sqrt{\sigma_{\mathrm{Nu}}^{2}+{\left(0.5\;\sigma_{\mathrm{Da}}\right)}^{2}}$. For a power–law reference $y=A\;x^{p}$ (with p=1 for Darcy/Elder and p=1/2 for Forchheimer), the resulting 1σ envelope is shown as $y\to y\;\exp(\pm \sigma_{\eta })$ with $\sigma_{\eta }=\sqrt{\sigma_{\ln y}^{2}+{\left(p\;\sigma_{\ln x}\right)}^{2}}$, yielding the shaded lower-bound uncertainty bands in the Darcy and Forchheimer regions.

#### SI Datasets.

The corresponding Nu–$Ra^{*}$ datasets used in this study are provided as *SI Appendix* as Dataset_SX, where X∈{1,⋯,52}. These datasets were obtained from three sources: Datasets S1–S19 and S31–S38 were supplied by Bavandla and Srinivasan; Datasets S20 and S26–S30 were compiled using the StarryData repository (https://wpd.starrydata2.org/, accessed between September 2025 and January 2026); and Datasets S39–S52 are the authors’ own previously published open-access data. In addition, a meta file, Datasets_meta.csv, is provided; it contains the relevant dataset information, descriptions, and all numerical values listed in Tables 1–3. The code used to reproduce the main-text Figs. 2 and 3 is included as CombinedFigures.txt, and the code used to plot the individual datasets is provided as eyeballing.txt. To run these scripts, install the required Python dependencies, place all provided .csv files in the same directory, change the file extension from .txt to .py, and execute the scripts.

## Supplementary Material

Appendix 01 (PDF)

Dataset S01 (TXT)

Dataset S02 (CSV)

Dataset S03 (CSV)

Dataset S04 (CSV)

Dataset S05 (CSV)

Dataset S06 (CSV)

Dataset S07 (CSV)

Dataset S08 (CSV)

Dataset S09 (CSV)

Dataset S10 (CSV)

Dataset S11 (CSV)

Dataset S12 (CSV)

Dataset S13 (CSV)

Dataset S14 (CSV)

Dataset S15 (CSV)

Dataset S16 (CSV)

Dataset S17 (CSV)

Dataset S18 (CSV)

Dataset S19 (CSV)

Dataset S20 (CSV)

Dataset S21 (CSV)

Dataset S22 (CSV)

Dataset S23 (CSV)

Dataset S24 (CSV)

Dataset S25 (CSV)

Dataset S26 (CSV)

Dataset S27 (CSV)

Dataset S28 (CSV)

Dataset S29 (CSV)

Dataset S30 (CSV)

Dataset S31 (CSV)

Dataset S32 (CSV)

Dataset S33 (CSV)

Dataset S34 (CSV)

Dataset S35 (CSV)

Dataset S36 (CSV)

Dataset S37 (CSV)

Dataset S38 (CSV)

Dataset S39 (CSV)

Dataset S40 (CSV)

Dataset S41 (CSV)

Dataset S42 (CSV)

Dataset S43 (CSV)

Dataset S44 (CSV)

Dataset S45 (CSV)

Dataset S46 (CSV)

Dataset S47 (CSV)

Dataset S48 (CSV)

Dataset S49 (CSV)

Dataset S50 (CSV)

Dataset S51 (CSV)

Dataset S52 (CSV)

Dataset S53 (CSV)

Dataset S54 (CSV)

Dataset S54 (TXT)

## Data Availability

All study data are included in the article and/or supporting information. Previously published data were used for this work (This study uses previously published datasets from Bavandla and Srinivasan (18, 19), Combarnous and Bories (15), Elder (16), Keene and Goldstein (24), Kladias and Prasad (22), Liu et al. (5), Schneider (20), and Schwendener et al. (23). All datasets used are shared in supporting information and available as cited in the manuscript. No new datasets were generated for this work.).
