# Supplementary material for: Dynamic confinement controls the porous-to-free convection transition
Source: Proc Natl Acad Sci U S A. 2026 May 28;123(22):e2533675123. doi: 10.1073/pnas.2533675123 (PMC13229175; doi:10.1073/pnas.2533675123)
Supplement: Supplementary file 1 — Appendix 01 (PDF) [file pnas.2533675123.sapp.pdf]

Supporting Information for

Dynamic confinement controls the porous-to-free convection transition

Dario M. Schwendener, J. Noir, J. Latt, C. Coreixas and X.-Z. Kong

Dario M. Schwendener (dario.schwendener@eaps.ethz.ch)
X.-Z. Kong (xiangzhao.kong@eaps.ethz.ch)

This PDF file includes:

Supporting text
Tables S1 to S3
SI References

Supporting Information Text

Here, we provide the extended versions of the tables shown in the main text (Tables S1, S2, and S3). Compared with the main-text tables, these extended tables additionally report the full name of the datasets shared, the porosity, the porous-medium Prandtl number Pr_p, and, for the experimental datasets, the ratio of domain height to bead diameter, H/d.

Table S1. Recent laboratory HRL datasets (2015–2025). Abbreviations: Ar = argon; Gl = glass; St = steel; Al = aluminium; PP = polypropylene; ϕ = porosity; H/d = height-to-bead-diameter ratio; Γ = confinement parameter; Pr = Prandtl number; Pr_p = porous-medium Prandtl number; k_m/k_f = solid-to-fluid thermal conductivity ratio; Da = Darcy number; Ra_c^* = critical modified Rayleigh number. The first column lists the corresponding dataset file names.

Dataset (.csv)	Fluid	Solid	ϕ	H/d	Γ	Pr	Pr_p	k_m/k_f	Da [$\times10^{-6}$]	Ra_c^*
Dataset_S1 (1)	Ar	Gl	0.402	25	0.001043	0.68	65	17.6	1.94	$4\pi^2$
Dataset_S2 (1)	Ar	Gl	0.409	50	0.000573	0.69	124	15.7	0.522	$4\pi^2$
Dataset_S3 (1)	Ar	Gl	0.389	66.7	0.000342	0.69	154	18.5	0.237	$4.34\pi^2$
Dataset_S4 (1)	Ar	Gl	0.396	83.3	0.000263	0.69	187	18.1	0.163	$5.14\pi^2$
Dataset_S5 (1)	Ar	Gl	0.401	100	0.000202	0.69	211	18.6	0.120	$6.25\pi^2$
Dataset_S6 (2)	Ar	Al	0.399	50	0.000367	0.70	61	34.3	0.469	$4\pi^2$
Dataset_S7 (2)	Ar	Al	0.407	150	0.000143	0.68	196	27.5	0.0568	$4\pi^2$
Dataset_S8 (2)	Ar	Gl	0.410	150	0.000199	0.69	366	14.6	0.0587	$4\pi^2$
Dataset_S9 (2)	Ar	St	0.401	50	0.000403	0.70	71	29.2	0.479	$4\pi^2$
Dataset_S20 (3)	Ar	PP	0.480	11	0.006326	0.69	108	5.04	21.8	$4\pi^2$

Table S2. Historic HRL datasets. Abbreviations: W = water; Gly = glycol; Trp = turpentine; Gl = glass; Acr = acrylic; St = steel; Pb = lead; Qz. S. = quartz sand; ϕ = porosity; H/d = height-to-bead-diameter ratio; Γ = confinement parameter; Pr = Prandtl number; Pr_p = porous-medium Prandtl number; k_m/k_f = solid-to-fluid thermal conductivity ratio; Da = Darcy number; Ra_c^* = critical modified Rayleigh number. The first column lists the corresponding dataset file names.

Dataset (.csv)	Fluid	Solid	ϕ	H/d	Γ	Pr	Pr_p	k_m/k_f	Da [$\times10^{-6}$]	Ra_c^*
Dataset_S10 (4)	Oil	Gl	0.351	26.8	0.002343	123.08	43247	1.72	0.957	$4\pi^2$
Dataset_S11 (4)	Oil	Gl	0.359	59.4	0.001106	123.08	95537	1.71	0.212	$4\pi^2$
Dataset_S12 (4)	W	Gl	0.371	13.4	0.006790	4.83	4706	1.03	4.81	$4\pi^2$
Dataset_S13 (4)	W	Gl	0.381	17.8	0.005389	4.83	6173	1.03	3.03	$4\pi^2$
Dataset_S14 (4)	W	Gl	0.385	31.5	0.003126	4.83	10810	1.03	1.02	$4\pi^2$
Dataset_S15 (4)	W	Pb	0.370	13.4	0.004753	4.83	2343	2.08	4.76	$4\pi^2$
Dataset_S16 (4)	Oil	Qz. S.	0.350	28.2	0.001993	123.08	36995	2.12	0.853	$4\pi^2$
Dataset_S17 (4)	W	Qz. S.	0.335	23.8	0.002267	4.83	5087	1.92	1.00	$4\pi^2$
Dataset_S18 (4)	W	Qz. S.	0.350	28.2	0.002083	4.83	5101	1.94	0.853	$4\pi^2$
Dataset_S19 (5)	W	Gl	0.400	10.0	0.009219	6.90	5959	1.15	9.90	$4\pi^2$
Dataset_S21 (6)	W	Acr	0.395	6.0	0.021155	5.71	7968	0.57	25.8	$4\pi^2$
Dataset_S22 (6)	W	Gl	0.375	25.4	0.002891	5.48	7761	1.37	1.16	$4\pi^2$
Dataset_S23 (6)	W	Gl	0.396	12.7	0.006529	5.40	4069	1.35	5.83	$4\pi^2$
Dataset_S24 (6)	Gly	Gl	0.400	25.4	0.002579	112.8	83949	2.27	1.53	$4\pi^2$
Dataset_S25 (6)	W	St	0.444	4.8	0.009086	5.88	702	5.88	68.5	55
Dataset_S26 (6)	W	St	0.388	12.0	0.002784	5.64	748	7.69	6.04	$4\pi^2$
Dataset_S31 (7)	Trp	Gl	0.393	4.0	0.013492	20.91	1518	3.12	57.5	$4\pi^2$
Dataset_S32 (7)	Trp	Gl	0.383	36.4	0.001399	20.91	12812	3.12	0.619	$4\pi^2$
Dataset_S33 (7)	W	Gl	0.400	4.0	0.022400	6.15	1407	1.22	61.9	$4\pi^2$
Dataset_S34 (7)	W	Gl	0.387	5.6	0.014840	6.15	1942	1.22	27.2	$4\pi^2$
Dataset_S35 (7)	W	Gl	0.388	10.0	0.008352	6.15	3324	1.22	8.62	$4\pi^2$
Dataset_S36 (7)	W	Gl	0.379	17.4	0.004595	6.15	5770	1.22	2.61	$4\pi^2$
Dataset_S37 (7)	W	Gl	0.377	36.4	0.002168	6.15	12135	1.22	0.581	$4\pi^2$
Dataset_S38 (7)	Trp	St	0.377	8.33	0.002648	20.91	612	15.62	11.1	$4\pi^2$

Table S3. Numerical HRL datasets. Abbreviations: FDM = finite difference method; LBM = lattice Boltzmann method; ϕ = porosity; Γ = confinement parameter; Pr = Prandtl number; Pr_p = porous-medium Prandtl number; k_m/k_f = solid-to-fluid thermal conductivity ratio; Da = Darcy number; Ra_c = critical Rayleigh number. Onset values are fluid Rayleigh numbers reported as $Ra_c\,[\times 10^6]$. The first column lists the corresponding dataset file names.

Dataset (.csv)	Type	ϕ	Γ	Pr	Pr_p	k_m/k_f	$Da\,[\times 10^{-6}]$	$Ra_c\,[\times 10^6]$
Dataset_S27 (8)	FDM	0.92	0.059400	4.3	11222	1.00	1300	0.112
Dataset_S28 (8)	FDM	0.87	0.040590	4.3	13123	1.00	450	0.238
Dataset_S29 (8)	FDM	0.82	0.028020	4.3	10845	1.00	180	0.498
Dataset_S30 (8)	FDM	0.75	0.018920	4.3	9237	1.00	75	1.09
Dataset_S39 (9)	LBM	0.33	0.001430	1.0	817	3.77	0.905	192.0
Dataset_S40 (9)	LBM	0.33	0.001674	1.0	1130	2.73	0.905	139.0
Dataset_S41 (9)	LBM	0.33	0.002770	1.0	3080	1.00	0.905	50.8
Dataset_S42 (9)	LBM	0.33	0.004553	1.0	8290	0.37	0.905	18.8
Dataset_S43 (9)	LBM	0.33	0.005333	1.0	11300	0.27	0.905	13.7
Dataset_S44 (9)	LBM	0.39	0.002626	1.0	838	3.22	2.62	56.5
Dataset_S45 (9)	LBM	0.39	0.003003	1.0	1100	2.46	2.62	43.2
Dataset_S46 (9)	LBM	0.39	0.004705	1.0	2700	1.00	2.62	17.6
Dataset_S47 (9)	LBM	0.39	0.007357	1.0	6520	0.41	2.62	7.20
Dataset_S48 (9)	LBM	0.39	0.008328	1.0	8490	0.32	2.62	5.62
Dataset_S49 (9)	LBM	0.43	0.003956	1.0	1010	2.84	5.25	24.9
Dataset_S50 (9)	LBM	0.43	0.004116	1.0	1270	2.62	5.25	23.0
Dataset_S51 (9)	LBM	0.43	0.006670	1.0	2870	1.00	5.25	8.76
Dataset_S52 (9)	LBM	0.43	0.009946	1.0	6370	0.45	5.25	3.94

References

1. KC Bavandla, V Srinivasan, Rayleigh–bènard convection in a gas-saturated porous medium at low darcy numbers. *ASME J. Heat Mass Transf.* **146**, 051001 (2024).
2. K Bavandla, V Srinivasan, Effects of solid-to-fluid conductivity ratio on thermal convection in fluid-saturated porous media at low darcy number. *ASME J. Heat Mass Transf.* **147** (2025).
3. DJ Keene, R Goldstein, Thermal convection in porous media at high rayleigh numbers. *J. Heat Transf.* **137** (2015).
4. M Combarnous, S Bories, Hydrothermal convection in saturated porous media in *Advances in hydroscience*. (Elsevier) Vol. 10, pp. 231–307 (1975).
5. JW Elder, Steady free convection in a porous medium heated from below. *J. Fluid Mech.* **27**, 29–48 (1967).
6. N Kladias, V Prasad, Experimental verification of darcy-brinkman-forchheimer flow model for natural convection in porous media. *J. thermophysics heat transfer* **5**, 560–576 (1991).
7. K Schneider, Investigation of the influence of free thermal convection on heat transfer through granular material. *Int. Inst. Refrig. Proc.* **247**, 253 (1963).
8. S Liu, et al., From rayleigh–bénard convection to porous-media convection: how porosity affects heat transfer and flow structure. *J. fluid mechanics* **895** (2020).
9. D Schwendener, J Noir, J Latt, C Coreixas, XZ Kong, Natural convection in porous media: the role of porosity and conductivity ratios in the transition from laminar to inertial convection. *J. Fluid Mech.* **1026**, A21 (2026).
